# Testicular mesothelioma disguised as hydrocele: a case report

**DOI:** 10.1186/s13256-024-04348-y

**Published:** 2024-02-27

**Authors:** Tanya Nazar, Anupama Gopalakrishnabhaktan, Fatema Ali Asgar Tashrifwala, Aroma Sathish, Tirth Dave

**Affiliations:** 1 VPS Lakeshore Hospital & Research Centre, Kochi, Kerala India; 2 Stamford Health, Stamford, USA; 3 Bukovinian State Medical University, Chernivtsi, Ukraine

**Keywords:** Testicular tumours, Hydrocoele, Mesothelioma, Pathology, Hydrocelectomy

## Abstract

**Background:**

Testicular tumors have many different manifestations. The majority of these cases are presented as an incidental finding during hydrocelectomy. Malignant mesotheliomas are uncommon tumours that can arise from the coelomic epithelium of the pleura, peritoneum, pericardium, and tunica vaginalis.

**Case presentation:**

We present a 51-year-old South Asian (Indian) male patient with a rare case of mesothelioma, presenting with right hydrocele, to whom a right hydrocelectomy was performed. Any history of trauma or asbestos exposure was not present. Histopathological and immunohistochemistry reports revealed a malignant mesothelioma of tunica vaginalis. There was no invasion of the tumour to the epididymis and spermatic cord. Imaging studies showed no signs of metastasis. 1 month later, a high inguinal orchidectomy was performed. The patient underwent adjuvant chemotherapy thereafter and is still on follow-up.

**Conclusion:**

Although hydrocele is common, detailed evaluation is mandatory to rule out certain rare tumours-testicular and paratesticular variants.

## Background

Hydrocele is a term for fluid collection in the scrotum. Commonly seen in men older than 40 years, it is a benign condition that can be surgically managed. However, acquired hydrocele is usually idiopathic and can be triggered by trauma, injections, and rarely testicular tumours [1]. Physical examination, radiological evaluation, and levels of testicular markers (alpha-fetoprotein, beta HCG, and LDH) are important to assist in diagnosis.

Mesothelioma of the tunica vaginalis is a rare variant that represents only 0.3–5% of all mesotheliomas [2]. Due to its rarity, epidemiology, and risk factors are still unclear, and it is unknown whether asbestos or chronic inflammatory conditions play a role in etiology [3]. Despite aggressive surgical procedures or adjuvant chemotherapy, the prognosis remains poor. Herein, we present an extremely rare case of right testicular hydrocele which was subsequently diagnosed as testicular mesothelioma, from pathological report.

## Case presentation

**Fig. 1:**
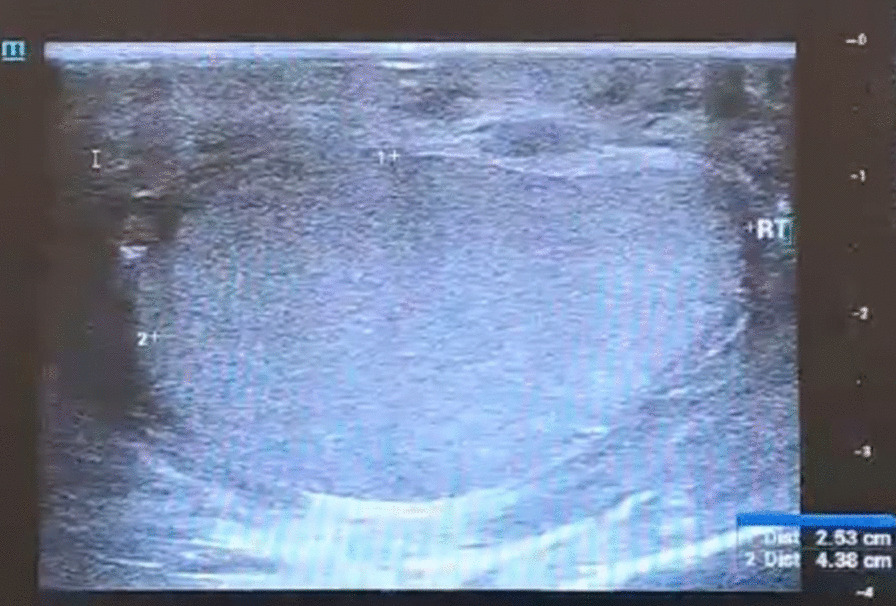
Initial USG

**Fig. 2:**
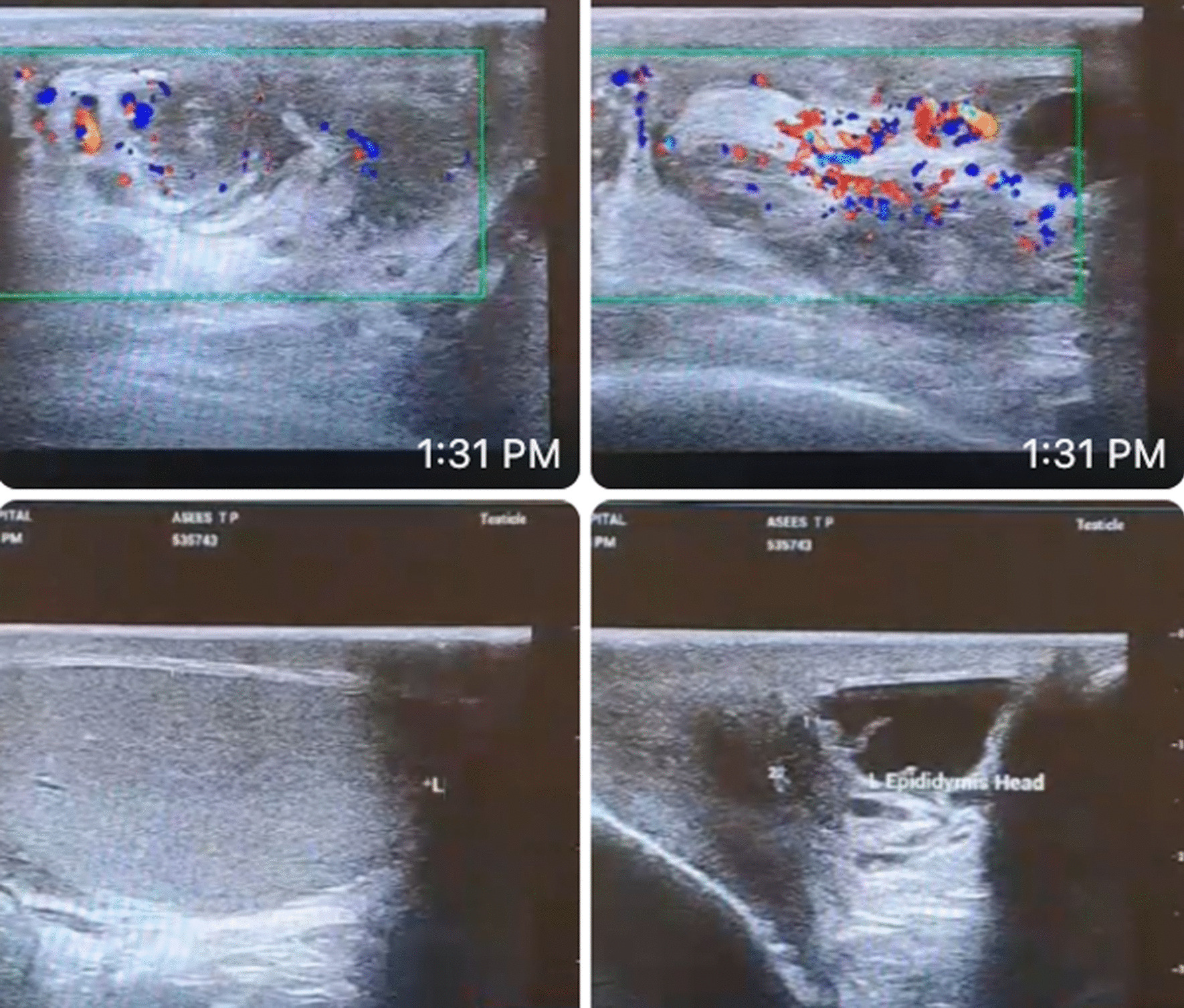
USG of the abdomen-post operative

**Fig. 3:**
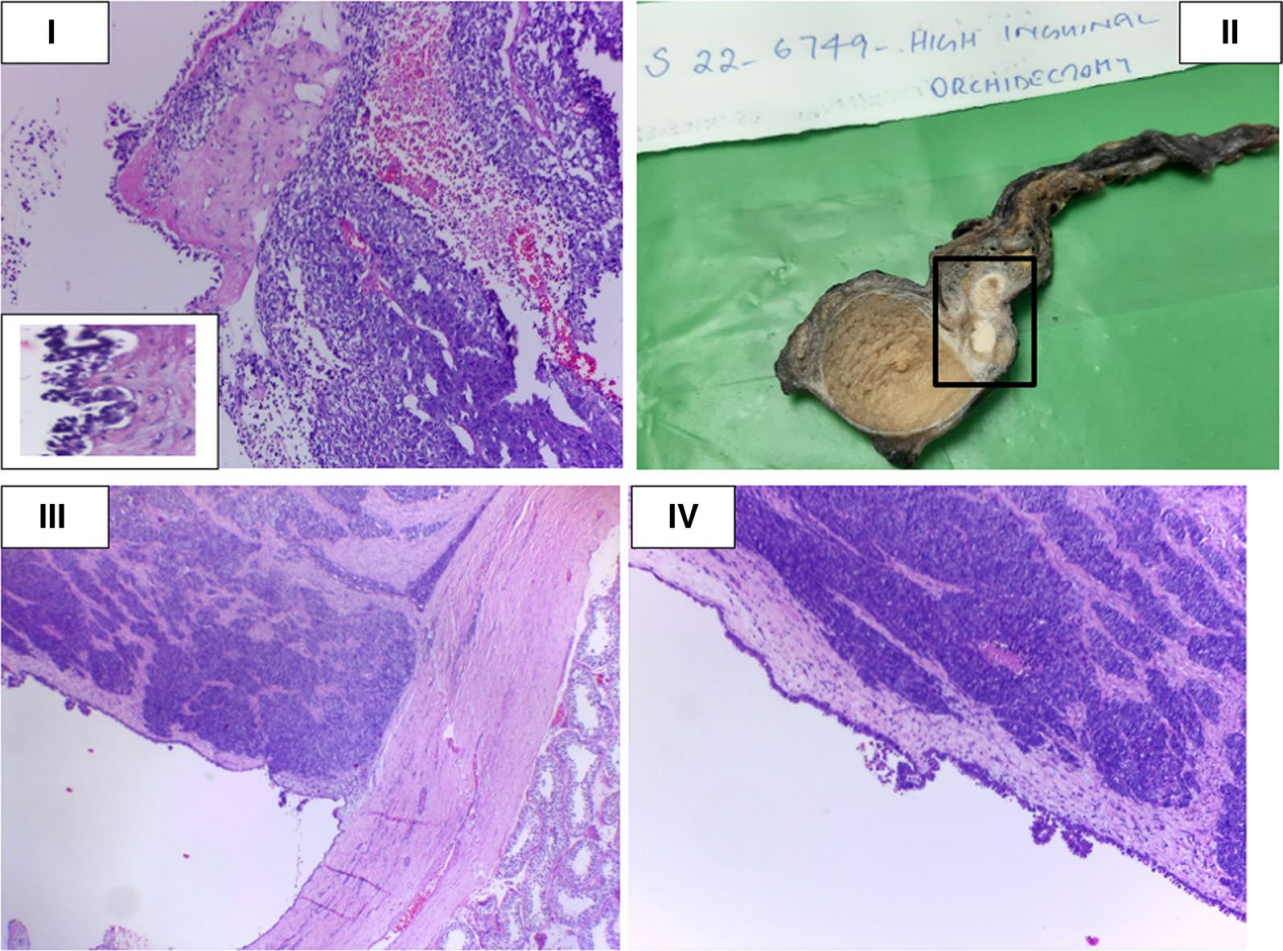
Microscopic image

**Fig. 4:**
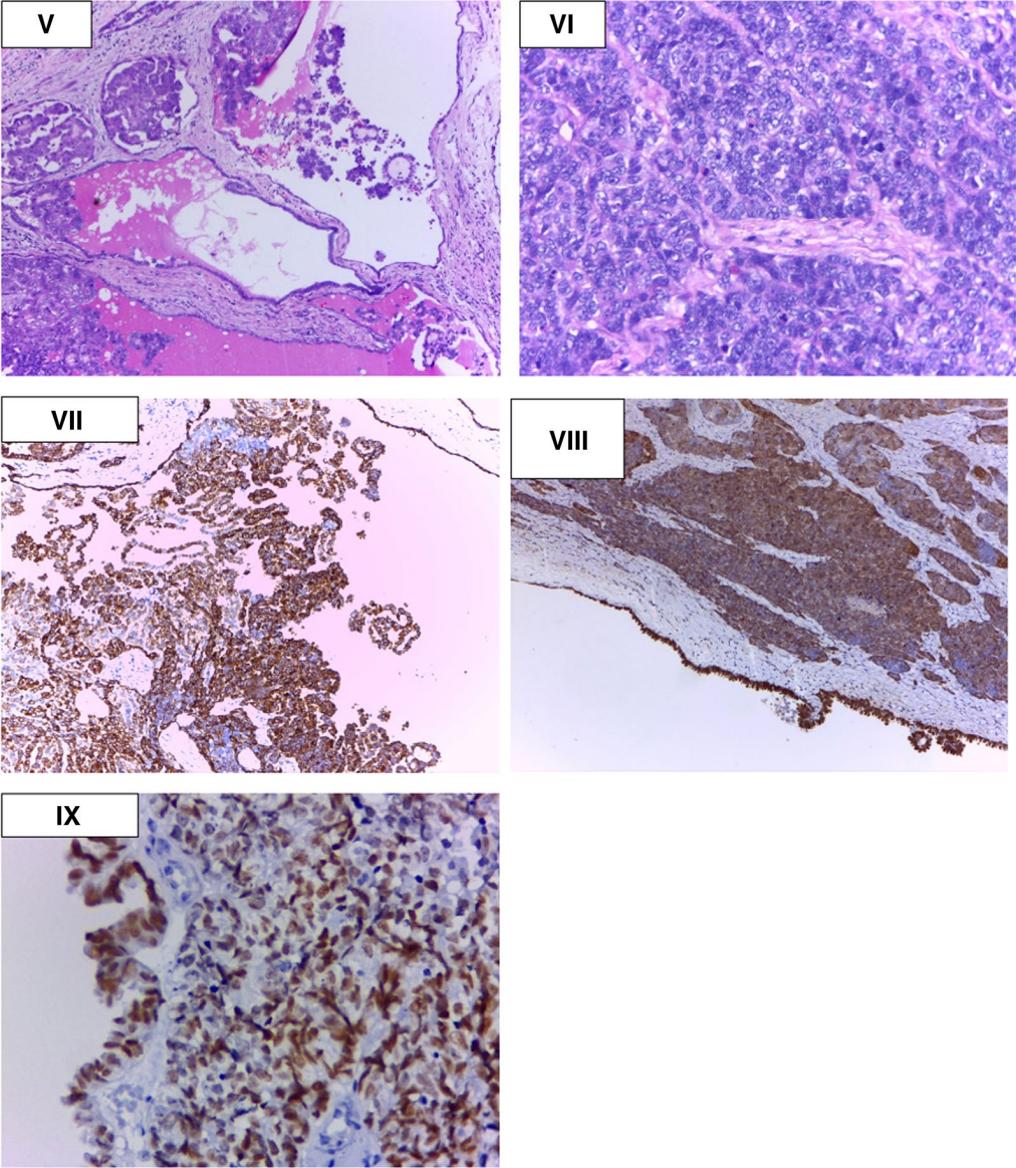
Microscopic image and immunohistochemistry

A 51-year-old South Asian (Indian) male patient presented to a peripheral hospital with right scrotal swelling of 1 year duration. The swelling has been increasing in size for 6–7 months. There is no relevant family, psychosocial, or surgical history. Past medical history is significant for diabetes mellitus for 5 years, controlled with medication (Metformin 400 mg), and negative for urinary incontinence, sexual disturbances, or hypertension. The patient does not smoke cigarettes or consume alcohol. On physical examination, a firm painless mass was palpable in the right scrotum suggestive of hydrocele with no palpable lymphadenopathy or inguinal hernias. Left testes and scrotum normal. Ultrasound findings showed features of chronic epididymitis, associated with an ill-defined heterogeneous area adjacent to the epididymis with no significant vascularity as seen in Fig. 1. Following this, CT abdomen was reported as peripherally enhancing fluid collection of size 2.9 × 2.2 cm in the right scrotum, by USG correlation, thick-walled septated collection in the right scrotum and right external iliac lymph node of size 1. I × 0.8 cm. Multiple calculi were seen in both kidneys, the largest measuring 6 mm on the right side and 8.2 mm on the left side. A 3.2 mm cyst was seen in the lower pole of the left kidney. There was no significant para-aortic lymphadenopathy and normal CT appearance of the liver, gall bladder, spleen, pancreas, adrenal glands, and urinary bladder was seen. There was no focal lesion to suggest metastasis. The pre-operative LDH, AFP, and beta-HCG levels were normal as seen in Table 1. These findings were suggestive of hydrocele and the patient underwent a right hydrocelectomy because of the rapid change in the size of the mass. Post-operative USG is seen in Fig. 2. During the surgery, a growth was found adhering to tunica vaginalis, and the same was excised along with the hydrocele sac. The growth was histologically confirmed as a neoplasm (Fig. 3(I)) and therefore high inguinal orchidectomy was done subsequently at our center. The specimen measured 7.5 × 5.5 × 4 cm with an attached spermatic cord measuring 11 cm in length (Fig. 3(II)). The cut section showed a residual grey-white tumour in the Para testicular soft tissue at the hilum, measuring 1.3 × 0.9 × 0.8 cm in size. Sections from the hydrocele sac showed a fibromuscular wall lined by atypical cells with papillary tufting (Fig. 3(I), inset) along with fragments of a poorly differentiated tumour arranged in solid nests and papillary pattern. Microscopically, the tumour contained papillary structures with confluent sheets and nests of tumour cells and necrotic areas. The tumour cells are crowded with indistinct cell borders, having clear to eosinophilic cytoplasm and vesicular pleomorphic nuclei with prominent nucleoli. Brisk mitosis was present (Figs. 3(III, IV) and 4(V, VI)). Immunohistochemistry was done to elucidate the nature of the tumour (Fig. 4(VII, VIII, IX)). A PET CT was done to rule out distant spread, suggesting the possibility of residual disease. Testis and epididymis were uninvolved.

**Table 1:** Initial blood test results

Test	Patient	Reference range
LDH	166 μ/L	140 to 280 (μ/L)
AFP	7.6 ng/mL	< 10 (ng/mL)
Beta HCG	0.1mIμ/L	< 2 mIμ/mL (in men)
Hb	13.1 g/L	13.8 to 17.2 (g/L)
Total WBC count	7690	4000–11,000 (cells/μL)
BUN/Cr	22:0.8	10:1
ALP	285	20 to 140 U/L

LDH: Lactate dehydrogenase; AFP: Alpha-Fetoprotein; Beta HCG: Beta human chorionic gonadotropin; Hb: Hemoglobin; Total WBC count: Total White Blood Cell count; BUN/Cr: Blood Urea Nitrogen to Creatinine ratio; ALP: Alkaline phosphatase

Immunohistochemical studies showed positive staining of the tumour cells for CK, CK7 (Fig. 4(VII)), Calretinin (Fig. 4(VIII)) and WT1 (Fig. 4(IX)). CK20, Inhibin, CD30, and Glypican 3 were negative. The pathology report indicated malignant mesothelioma of the right tunica vaginalis, a poorly differentiated variant as seen in Figs. 3 and 4. Although, the lymphovascular invasion was present, epididymis, testes, and spermatic cord showed no infiltrates. The patient was given 6 cycles of pemetrexed and carboplatin once in 3 weeks. Reassessment imaging studies showed a complete treatment response. Therefore, we have been following up with the patient in our OPD to date as seen in Table 2. All follow-up visits have shown no clinical symptoms of tumour growth, and no indication is seen on radiological investigations.

**Table 2:** Latest blood test reports-post treatment

Test	Patient	Reference range
LDH	Not measured	140 to 280 (μ/L)
AFP	Not measured	< 10 (ng/mL)
Beta HCG	Not measured	< 2 mIμ/mL (in men)
Hb	12.7 g/L	13.8 to 17.2 (g/L)
Total WBC count	7400	4000–11,000 (cells/μL)
ALT	93	7–55 U/L
BUN/Cr	Not measured	10:1
ALP	155 U/L	20 to 140 μ/L

LDH: Lactate dehydrogenase; AFP: Alpha-Fetoprotein; Beta HCG: Beta human chorionic gonadotropin; Hb: Hemoglobin; Total WBC count: Total White Blood Cell count; ALT: Alanine aminotransferase; BUN/Cr: Blood Urea Nitrogen to Creatinine ratio; ALP: Alkaline phosphatase

## Discussion

Malignant mesothelioma of paratesticular origin is a rare tumor, representing only 0.3–5% of all mesotheliomas. It arises from the tunica vaginalis, which forms a double layer of mesothelium lining the outer surface of the tunica albuginea and the inner layer of the scrotum. It can affect males of varying ages but is most common in those aged between 55 and 75 years [4]. Interestingly, the incidence rate increases with age, with males over the age of 80 having significantly higher rates [5]. Exposure to asbestos is a well-established risk factor for pleural and peritoneal mesotheliomas, its link to tunica vaginalis tumors remains unclear. Studies of asbestos-exposed occupational cohorts have reported no cases of malignant testicular mesothelioma. Other suspected risk factors include trauma, long-term hydrocele, epididymitis, orchitis, or other inflammatory conditions. Long-standing hydrocele has been associated with an increased risk of developing testicular mesothelioma [5] [6]. Diagnosing this condition pre-operatively can be challenging due to the lack of specific clinical or radiological features. Physical examination and radiography are generally used to detect lesions, and ultrasonography is a noninvasive and accurate method to detect testicular tumors, aiding in differential diagnosis [7]. Testicular tumor markers can also help in the diagnosis of germ cell tumors. Immunohistochemically, these tumors exhibit similarities to pleural mesotheliomas with diffuse positivity for CK7, Calretinin, and WT1. However, Calretinin expression should be interpreted in the context of a broader panel including CK5/6, WT1, and podoplanin [8]. The emerging marker HEG1 is reportedly 100% specific for epithelioid mesothelioma. Unfortunately, the treatment of malignant mesothelioma of paratesticular origin has yielded disappointing results. Tumors tend to recur within 1–2 years, with common sites of metastasis including retroperitoneal/inguinal lymph nodes, the brain, lung, and bone. Biphasic subtypes, higher disease stage, and a critical tumor size cutoff of 4 cm have been reported as poor prognostic features associated with poor survival [9].

Presentation as hydrocele is common, resulting in gross thickening of the tunica vaginalis forming white/tan, solid/papillary masses enveloping the testicular parenchyma and associated with fluid in the hydrocele sac [10]. Invasion into the testicular parenchyma is also frequently observed. Histologically, several patterns are described, with the most common being the epithelioid type with papillary or tubulopapillary architecture. Sarcomatoid and biphasic variants are rare. Occasionally, an in-situ component with atypical mesothelial cells lining the tunica vaginalis can be seen, which is a valuable clue in arriving at the correct diagnosis. Treatment options for testicular mesothelioma may include surgery, radiation therapy, and chemotherapy. In cases of localized disease, radical inguinal orchidectomy is the primary surgical approach, with the addition of inguinal lymph node dissection if metastasis is present. The use of adjuvant chemotherapy and/or radiotherapy is still under investigation, with cisplatin and pemetrexed being potential chemotherapeutic options [11]. Due to the disease's poor prognosis, adjuvant chemotherapy is advisable. The mortality rate from testicular mesothelioma is high, with over 50% of patients experiencing local or distant recurrence, often within the first 2 years [6]. Long-term follow-up is essential, as recurrence can occur even after many years [11].

## Conclusion

In summary, we present a case of a patient with testicular mesothelioma of tunica vaginalis. The rarity of testicular mesothelioma poses challenges to its etiology research, diagnosis, and treatment. Diagnosis of testicular mesotheliomas is challenging, as the tumour lacks specific clinical and radiologic features, and the reported sex cord-like pattern proves its histological diversity. Despite aggressive surgical procedures or extra testicular mesothelioma-based adjuvant therapies, the prognosis remains poor. In conclusion, a hydrocele should be closely monitored. Testicular mesothelioma is extremely rare but should be kept in mind when diagnosing patients with a testicular mass, even if they have no history of exposure to a risk factor.

## Data Availability

Available upon reasonable request from the corresponding author.
